# Book Notices

**Published:** 1895-03

**Authors:** 


### Book Notices.

DISEASES OF THE CHEST, THROAT, AND NASAL CAVITIES. Including Physical Diagnosis and diseases of the Lungs, Heart, and Aorta, Laryngology and Diseases of the Pharynx, Larynx, Nose, Thyroid Gland, and Œsophagus. Third edition, revised. With 240 illustrations, 8vo, 718 pages. By E. Fletcher Ingals, A. M., M. D., Professor of Laryngology and Practice of Medicine, Rush Medical College; Professor of Diseases of the Throat and Chest, Northwestern University Woman's Medical School, etc., etc. Price, muslin, $5. William Wood & Co., Publishers, 43, 45 and 47 East Tenth Street, New York City.

The second edition of Dr. Ingalls' book met with a cordial reception by the medical profession generally, and he has found it necessary to issue a third edition in less than two years from the time the second was printed. In this edition, no great alteration has been made in the text, but several minor changes have been made, and a few pages have been added, to keep abreast of our advancing knowledge on the subjects under discussion.

The subjects of the etiology, pathology, symptomatology, diagnosis, prognosis, and treatment of the diseases of the respiratory and circulatory organs, are here presented in a convenient form.

The volume contains thirty-seven chapters, fifteen of which are devoted to the consideration of diseases of the chest, fourteen to that of diseases of the throat, seven to diseases of the nose, and one to diseases of the thyroid gland and œsophagus.

Of the fourteen chapters on diseases of the chest, four are devoted to the consideration of physical diagnosis, six to pulmonary diseases, two to the heart, two to cardiac diseases, and one to diseases of the thoracic arteries. The fourteen chapters on diseases of the throat are allotted as follows: Two to the throat and its examination, four to diseases of the fauces, one to diseases of the pharynx, and seven to diseases of the larynx. Of the seven chapters treating of diseases of the nose, six are on diseases of the nasal cavities, and one on diseases of the naso-pharynx.

The book contains an appendix, giving many valuable formulæ for gargles, trochisci, vapor inhalations, spray inhalations, dry inhalations, pigments, insufflations, and nasal douches.

The general arrangement of the book, the amount of space allotted to each subject, and the thorough manner in which each subject is discussed, all contribute to its superior worth. One of its many valuable features is the parallel tables in making differential diagnosis. The author's experience as a teacher of practice of medicine and laryngology has rendered him conversant with the needs of the student and practitioner, and has enabled him to produce a book of the greatest interest and utility to them. H.

---

LECTURES ON AUTO-INTOXICATION IN DISEASE, or Self-Poisoning of the Individual. By Ch. Bouchard, Professor of Pathology and Therapeutics, Member of the Academy of Medicine, and Physician to the Hospitals, Paris. Translated, with a preface, by Thomas Oliver, M. A., M. D., F. R. C. P., Professor of Physiology, University of Durham; Physician to the Royal Infirmary, Newcastle-upon-Tyne, and Examiner in Physiology, Conjoint-Board of England. In one octavo volume; 302 pages. Extra cloth, $1.75 net. Philadelphia: The F. A. Davis Co., publishers, 1914 and 1916 Cherry street.

The thirty-two lectures in this volume treat of the operation of poisons introduced from without or generated within the body of men, and the part they play in health and disease. They deal with the production and elimination of poisons by the organisms; the toxicity of urine, the part the toxic principles in urine play in producing uræmia; toxicity of the blood and tissues; toxicity of the fluids and of the contents of the intestines (bile and the products of putrefaction); intestinal antisepsis; pathogenesis of uræmia, distinction between the symptoms of the pre-uræmic period of nephritis and the symptoms of intoxication; the part played by organic substances and mineral matters in uræmic intoxication, the therapeutic pathogenesis of uræmia; transitory or acute auto-intoxication of intestinal origin—internal strangulation and constipation—gastric disorders—indigestion—poisoning by tainted meats; chronic gastro-intestinal auto-intoxication dilation of the stomach—etiology, pathogenesis and therapeusis; auto-intoxication of intestinal origin; typhoid fever, pathogenic therapeusis of typhoid fever, antisepsis of the internal medium, rea tment of high temperature, new mode of bathing in fevers, dieting of fever patients; auto-intoxication of bile, pathogenesis of jaundice, malignant jaundice, aggravated jaundice; the toxic nature of pathological urines; pyocyanic disease; poisoning accidents in diabetes; poisoning by pathological poisons; cholera; and the general therapeutics of self-poisoning.

A careful study of this book will convince any one that auto-intoxication, or self-poisoning, is a much more important factor in the production of pathological conditions than is generally supposed, and that putrefactive processes in the alimentary canal and the development of physiological and pathological alkaloids play an important part in many diseased processes until lately unknown or misunderstood.

The practical application of the knowledge gained in this volume is in the prevention of the introduction of poisonous or putrefactive substances, the elimination of toxic matter, and the destruction of these poisonous principles in the economy by the administration of suitable antiseptic agents.

In the treatment of typhoid fever, for instance, the advice of the author to depend largely on the administration of antiseptics, is in accord with the latest teaching by the more progressive men of our time, and there can be no doubt that by this method the course of this disease (and many others) can be favorably influenced. The book is a most excellent one, and no physician can give it careful study and not be greatly benefited thereby.

H.

---

TWENTIETH CENTURY PRACTICE. An International Encyclopedia of Modern Medical Science. By Leading Authorities of Europe and America. Edited by Thomas L. Stedman, M. D., New York City. In Twenty Volumes. Volume I, Diseases of the Uropoietic System. Price, cloth, $5.00; leather, $6.00; half morocco, $7.50. New York: William Wood and Company. 1895.

This is the first volume of what may justly be termed the crowning medical publication of a century rich in the literature of the healing art. The last great work of this kind, devoted to internal medicine, that of Ziemssen, was published before the new science of bacteriology was developed; and it is fitting now that the publishers of the English translation of that work should bring out a new encyclopedia of modern medicine as it is at the end of the nineteenth and beginning of the twentieth centuries. As we learn from the announcement, the work will consist of twenty volumes, the first twelve being devoted to the systematic affections, including diseases of the skin and nervous system, and the remaining eight containing treatises on the infectious diseases. The writers have been chosen from all the countries of Europe, as well as from America, and are almost without exception, men of international reputation who have won for themselves a position in the first rank of medical teachers.

Volume I., which has just appeared, treats of disease of the uropoietic system. The first article, on diseases of the kidneys, is from the pen of Dr. Francis Delafield, of New York. The classification of kidney diseases, which the author makes, is extremely simple, and assists the reader greatly in arriving at a clear understanding of the morbid changes which these organs undergo. The diseases of the renal pelves, the ureters, and the bladder are presented in two excellent articles, by Mr. Reginald Harrison, London. These are followed by two systematic and lucid treatises on the diseases of the prostate and male urethra, by Dr. G. Frank Lydston, of Chicago. The diseases characterized by changes in the urine (hæmaturia, cystinuria, chyluria, pyuria, etc.) The albuminuria of nephritis and diabetes mellitus are not included in this article. The closing treatise of the volume is one on the diseases of the female bladder and urethra, by Dr. Howard A. Kelly, of Baltimore. In this article the author describes at length his new method of examination of the bladder and ureters in the female, which we believe has never before been described in any text-book or treatise.

---

A Manual of Modern Surgery, General and Operative. By John Chalmers Da Costa, M. D., Demonstrator of Surgery, Jefferson Medical College, Philadelphia; Chief Assistant Surgeon, Jefferson Medical College Hospital, etc., with 188 Illustrations in the text, and 13 full-page plates in colors and tints, aggregating 276 separate figures. Price in cloth, $2.50 net. W. B. Saunders, Publisher, 925 Walnut Street, Philadelphia. 1894.

This volume of 809 pages is not to be confounded with the incomplete compends of surgery, but is intended to occupy a place between these and the large and cumbrous textbooks. Its aim, as the author announces in his preface, is to present in clear terms and in concise form the fundamental principles, the chief operations, and the accepted methods of modern surgery; and in this purpose the author has succeeded most satisfactorily.

To operative surgery has been given more than ordinary space and attention, the most important procedures being fully described, giving also the instruments and appliances necessary, and the position assumed by patient and operator.

Fractures and dislocations, the most important part of surgical work, especially with the general practitioner, are here fully discussed, a large amount of space being devoted to their consideration.

Diseases and injuries of special parts and organs, the head, the heart, the bones and joints, the spine, the digestive tract, the abdomen, the rectum and anus, the muscles, tendons, nerves, etc., have received careful discussion.

Orthopedic surgery has received due attention, and surgical dressings, antiseptics, anæsthetics, etc., are given the attention their importance demands.

The book is thoroughly practical, and as concise as can be made to include the essentials in surgery. H.

---

BACON'S CIPHER STORY. By Dr. Orville W. Owen, Detroit, Mich., Howard & Co., Detroit, Publishers. Four Volumes, in neat covers, 50 cents per volume.

Dr. Owen, when a tardy and incredulous public shall have done him justice, will be immortal. The discovery of the cipher which for perhaps a hundred years has been believed to lie hidden somewhere in Bacon's writings, whereby the secret so securely locked up for three centuries is at last revealed; the deciphering of the wonderful, thrilling secret history of Elizabeth and her times—the revelation of the fact that Bacon was the rightful King of England—and the manner in which he was defrauded of his birth-right ought to constitute one of the most striking epochs of the nineteenth century, and live forever in history,—and it will. The importance of the discovery can scarcely be comprehended. Shakespeare is so enshrined in the hearts of all people that it will be hard to dislodge him; to make them realize that all the glory which has so long surrounded his name was but a borrowed light, and by rights belongs to another. But this, Dr. Owen has, in our judgment clearly demonstrated. It is utterly absurd to suppose for a moment that he could have manufactured the story; and had he done so, no mortal man could have put it into the language with which it is clothed. Dr. Owen asks no one to take his word for anything; he submits proof as strong as those from holy writ—in substantiation of every claim. In volume 4, just out, he reveals the secret which readers have been so long and impatiently expecting,—the *how* he found the key, and the manner of deciphering.

While a medical journal is not exactly the place for a review of a work of the kind, we make this mention here believing our readers will be interested in the subject, and will want to read the works. It is most intensely interestly, thrilling, astounding, and may well challenge belief. It will, no doubt, and should be, dramatised. D.

---

LANDMARKS IN GYNECOLOGY. By Byron Robinson, B.S., M.D., Chicago, Ills., Professor of Gynecology in the Chicago Post-Graduate School; Gynecologist to the Woman's Hospital, the Charity Hospital, and the Post-Graduate Hospital. In two volumes, of about 110 pages each. Price per volume, paper cover, 25 cents; cloth, 50 cents. George S. Davis, Publisher, Detroit, Michigan. 1894.

These little volumes are written in Dr. Robinson's usually happy and interesting style. They contain an abstract of some of his lectures at the Chicago Post-Graduate School. He divides the subject into prominent "Landmarks" for convenience of teaching, and for the purpose of impressing upon practitioners the chief features of gynecology.

His "Landmarks" are as follows: (1) Anatomy; (2) Menstruation; (3) Labor; (4) Abortion; (5) Gonorrhœa; (6) Tumors.

It is safe to say, says the author, that in one of these landmarks will be found the disease from which the patient suffers.

The author presents some original views and new classifications; and his writings give evidence of personal labor and investigation. H.

---

A MANUAL OF PRACTICE OF MEDICINE, PREPARED ESPECIALLY FOR STUDENTS. By A. A. Stevens, A. M., M. D., Lecturer on Terminology, and Instructor in Physical Diagnosis in the University of Pennsylvania; Demonstrator of Pathology in the Woman's Medical College, etc., etc. Third edition, revised. Illustrated. Price, cloth $2.50. W. B. Saunders, Publisher, 925 Walnut Street, Philadelphia.

This book was written for the especial use, and at the request of medical students. There is no denying the fact that the average text-book on the practice of medicine is confusing to the mind of the beginner in the study of medicine, because of the theoretical discussions in which it engages, and the very minute details it observes in presenting any subject to the student. In this condition of things, a more concise work, if proper care and judgment have been exercised in its preparation, will prove of greater value to the medical student than the very large text-books.

This book of Dr. Stevens has been prepared with the greatest care, and the very difficult task of leaving out that which is of the least importance, has been well performed. We take pleasure in recommending the book to medical students. H.

A TREATISE ON DIPHTHERIA. By Dr. H. Bourges. Translated by E. P. Hurd, M. D., Professor of Pathology in the College of Physicians and Surgeons, Boston, Mass., Physician to the Newberry Hospital, etc., etc. 173 pages. Price, paper cover 25 cents, in cloth 50 cents. George S. Davis, Publisher, Detroit, Mich. 1894.

Dr. Bourges has written a very interesting monograph on the important subject of diphtheria. He gives its history; etiology, bacteriology, symptoms, complications, progress, duration, termination, diagnosis, prognosis and treatment.

In all he has exercised care and judgement that the information furnished might be as complete, as fully up to date and as practical as possible. He has not attempted to give the innumerable variety of therapeutic methods and agents which have been proposed for diphtheria, but has confined his recommendations to such agents as have stood the test of continued trial. No doctor can read the book without profit. H.

---

ESSENTIALS OF DISEASES OF THE EAR. Arranged in the form of questions and answers. Prepared especially for students of medicine and post-graduate students. By E. B. Gleason, M. D., Clinical Professor of Otology, Medico-Chirurgical College, Philadelphia; Surgeon in charge of the Nose, Throat and Ear Department of the Northern Dispensary, Philadelphia. Price, in cloth, $1.00. W. B. Saunders, publisher, 925 Walnut St., Philadelphia, Pa.

This volume contains 147 pages, and is No. 24 of Saunders' Series of Question Compends. It was the intention of the author to supply a book mainly for physicians who may desire to take a post-graduate course in Otology. There can be no doubt that a book arranged in the form of questions and answers is of greater value to the physician who wishes to make a hurried review of the subject than is a large text-book. In a book of this kind and prepared with as much care and painstaking as this one has been, everything essential to the subject can be compressed into very small space. H.

---

SYLLABUS OF LECTURES ON HUMAN EMBRYOLOGY. An Introduction to the Study of Obstetrics and Gynecology. For Students and Practitioners. With a Glossary on Embryological Terms. By Walter Porter Manton, M. D., Professor of Clinical Gynecology and Lecturer on Obstetrics in the Detroit College of Medicine, etc., etc. Illustrated with numerous outline drawings and photo-engravings. Price, cloth, $1.25 net. The F. A. Davis Co., publishers, 1916 Cherry street, Philadelphia, Pa.

This book contains one hundred and twenty-five pages of reading matter and is interleaved for taking notes. The book will be valuable to both teachers and students. To the former it will furnish printed headings that may be used in the class room, serving as a guide during the elaboration of the subject by the teacher. To the student it will furnish an outline of the principal facts in human embryology, and the blank pages afford convenient space for taking notes of the lectures.

The book does not go into the details and theories of human embryology, but for these the student is referred to larger works dealing with this branch of medical science. The volume is a handsome one, and we believe will become quite popular. H.

---

ESSENTIALS OF DISEASES OF THE SKIN, INCLUDING THE SYPHILODERMATA. Arranged in the form of questions and answers. Prepared especially for students of medicine. By H. W. Stelwagon, M. D., Ph. D., Clinical Professor of Dermatology in Jefferson Medical College etc., etc, etc. Philadelphia: W. B. Saunders, 1894. $1.00.

This is the third edition of this book. It is clearly written, nicely illustrated and contains much that is of value, not only to the student but to the practitioner as well. Dr. Stelwagon is one of our ablest teachers of dermatology, and what he says on this subject may be considered as authority. H.

